# Smartphone-Based Activity Recognition in a Pedestrian Navigation Context

**DOI:** 10.3390/s21093243

**Published:** 2021-05-07

**Authors:** Robert Jackermeier, Bernd Ludwig

**Affiliations:** Chair for Information Science, University Regensburg, 93053 Regensburg, Germany; bernd.ludwig@ur.de

**Keywords:** activity recognition, smartphone, pedestrian navigation, naturalistic data, machine learning

## Abstract

In smartphone-based pedestrian navigation systems, detailed knowledge about user activity and device placement is a key information. Landmarks such as staircases or elevators can help the system in determining the user position when located inside buildings, and navigation instructions can be adapted to the current context in order to provide more meaningful assistance. Typically, most human activity recognition (HAR) approaches distinguish between general activities such as walking, standing or sitting. In this work, we investigate more specific activities that are tailored towards the use-case of pedestrian navigation, including different kinds of stationary and locomotion behavior. We first collect a dataset of 28 combinations of device placements and activities, in total consisting of over 6 h of data from three sensors. We then use LSTM-based machine learning (ML) methods to successfully train hierarchical classifiers that can distinguish between these placements and activities. Test results show that the accuracy of device placement classification (97.2%) is on par with a state-of-the-art benchmark in this dataset while being less resource-intensive on mobile devices. Activity recognition performance highly depends on the classification task and ranges from 62.6% to 98.7%, once again performing close to the benchmark. Finally, we demonstrate in a case study how to apply the hierarchical classifiers to experimental and naturalistic datasets in order to analyze activity patterns during the course of a typical navigation session and to investigate the correlation between user activity and device placement, thereby gaining insights into real-world navigation behavior.

## 1. Introduction

Accurate and actionable navigation instructions are a critical part of almost any navigation system involving humans. In the context of smartphone-based pedestrian navigation, instructions that take not just the user’s location but also their current activity into account can provide better assistance [1]. In fact, a user’s position and activity are often correlated with each other, e.g., when walking up stairs or through a doorway. In these cases, activities can act as landmarks that—combined with a model of the environment—help in pinpointing the user location [2,3].

Most HAR methods in the literature are geared towards activities of daily living (ADL), i.e., the detection of activities such as walking, standing, sitting, etc. While this information is certainly beneficial in a pedestrian navigation setting as well, we present in this work an approach to distinguish between more fine-grained activities that is tailored to the needs of pedestrian navigation. For example, a user who stops walking unexpectedly and then starts looking around may require additional information or navigation instructions in order to progress along the route. To our knowledge, HAR for these types of detailed navigation-related activities has not been investigated yet in the literature.

HAR on mobile devices is enabled by integrated MEMS sensors such as the accelerometer, gyroscope and magnetometer that measure various physical properties of the device, allowing the system to infer the user’s state or activity. Depending on user activity and device placement, different acceleration, orientation and rotation patterns can be detected and subsequently classified by ML algorithms. Traditional ML methods such as Support Vector Machines or Decision Trees are dependent on labor-intensive feature engineering and require domain knowledge by the researcher/engineer. In contrast, current deep learning-based methods are extracting the features directly from the data without the need for human intervention [4]. Challenges for smartphone-based HAR are posed by the varying availability and accuracy of built-in sensors as well as the many options for device placement that may even change over the time of a navigating session [5]. We try to overcome these issues by focusing on widely available sensors and by training dedicated models for different placements.

Since the first mobile phones with integrated inertial sensors became widely available more than 10 years ago, HAR has evolved rapidly, along with increased mobile computing power and improved sensor technology that allow on-device solutions to run in real-time [6]. In 2012, Wang et al. [2] detected elevators, staircases and escalators in the inertial sensor output with a decision tree containing hand-crafted features and thresholds [2]. Zhou et al. [7] also relied on a decision tree to distinguish among the activities walking, stairs up/down, elevator up/down, escalator up/down and keeping still [7]. Martinelli et al. [8] augmented dead reckoning applications with movement information by detecting changes in posture (e.g., sitting → standing) and cyclical activities (e.g., walking and climbing/descending stairs). Utilizing data from a belt-worn sensor, they evaluated various supervised learning methods such as Decision Trees, Support Vector Machines and K-Nearest Neighbor [8].

Traditional ML methods that rely on manual feature extraction in order to obtain competitive results have largely been surpassed by deep learning methods in recent years. Recurrent neural networks (RNNs) and especially long short-term memory (LSTM) cells as well as convolutional neural networks (CNNs) have proven successful at dealing with temporal patterns in input data. Wang et al. [9] provided an overview over the advantages of deep learning for HAR and the various approaches used throughout the literature [9]. As it is one of the most consistent and well-performing approaches in their comparative evaluation, the DeepConvLSTM method [10] serves as a baseline in our evaluation later on. Other examples for deep learning based HAR approaches for pedestrian navigation include those by Yang et al. [11] and Zhou et al. [12], who used a CNN to distinguish among nine activities with accuracies of about 95% and 98%, respectively. A comparison of traditional and deep learning methods for motion mode and smartphone posture recognition was conducted by Ye et al. [13]. Their LSTM- and CNN-based models reach a higher precision than several other traditional methods [13]. As in our work, the models are also applied afterwards to real-world scenarios and are ultimately designed to improve indoor positioning performance. In a recent work by Ebner et al. [14], more traditional methods such as codebooks, statistical features and analytical transformations are featured [14]. Similar to our work, the focus there lies on locomotion activities typical for indoor navigation.

The idea of hierarchical classification explored in this work was also investigated successfully by Zhang et al. [15] in the related context of ADL. They used rule-based reasoning to distinguish between motion and motionless activities followed by support vector machines to further discriminate the classes [15]. Another similar example can be found in [16] for the use-case of smartphone transportation mode recognition based on a two-layer framework that combines time-domain and frequency-domain data. After a multiclass classifier chooses the two most probable transportation modes, subsequent binary classifiers lead to a more robust result.

Some activities (e.g., standing still) can occur at almost any place, while others are tied to specific locations such as corners, doors or staircases. Gu et al. [17] demonstrated how the latter activities can be integrated into a pedestrian dead reckoning (PDR) based indoor localization system by combining them into a so-called landmark graph that is correlated with a floor plan [17].

To cover a wide range of applications, a smartphone-based HAR solution needs to support multiple device placements. Especially in the case of pedestrian navigation, the system cannot rely on the user always carrying their device in an identical manner. There are many examples in the literature where multiple device placements were considered: A lowest common denominator found in almost all approaches is the distinction between a handheld device and one carried inside a pocket [14,18]. These modes are often supplemented with additional placements or variations such as a waist-mounted device [19,20], a device swinging in the hand or arm [20,21,22,23,24] or held near the head such as during a phone call [20,22,24]. Less frequent placements include the device being strapped to the upper arm [25] or located in a backpack [20,23] or shirt pocket [25].

De-La-Hoz-Franco et al. [26] provided an overview over many HAR datasets presented in the literature. For the use-case of smartphone-based pedestrian navigation, datasets that were collected by means of additional sensors (e.g., foot-mounted or belt-worn) cannot immediately be applied. Furthermore, a dataset should ideally contain multiple types of locomotion and stationary behavior that allow not only to detect whether a user is walking or standing, but also to infer either a more precise location (e.g., staircases or elevators) or additional information about the user’s state. Examples for the latter are the distinction between walking and running to establish whether the user is in a hurry or—as we show below in our dataset—the inclusion of different stationary modes. Finally, some activities such as sitting and lying are mainly of interest in ADL, but do not often occur in pedestrian navigation. In fact, most of the existing datasets are focused on ADL and do not meet these requirements. There are a few exceptions worth noting that almost fit our needs: in the dataset presented in [27], the activities are walking, upstairs, downstairs, standing, sitting and lying. Unfortunately, the data were collected using a waist-mounted sensor only and therefore is not applicable to smartphone-based navigation. The UniMiB SHAR dataset [28] contains the activities walking, running, jumping, falling, sitting, lying, going up/down and hitting obstacle. Only an accelerometer was used as a single sensor for data collection. Both datasets consist of relatively generic categories that cannot provide a lot of additional information about the user’s current state.

To conclude, while there are many HAR approaches available in the literature, the amount specifically geared towards pedestrian navigation and focused on gaining additional information to be exploited by an assistance system is quite low and warrants further investigation. As established above, many HAR approaches rely on basic activities such as walking, standing, sitting and—in some circumstances—walking up or down stairs. For the use case of pedestrian (indoor) navigation, other activities are relevant as well, e.g., taking an elevator or opening and closing doors. In this work, we are interested in even more granular activities and try to differentiate among the following:

(a) a stationary user who is standing still, e.g., while reading signage on a wall or instructions on their phone;
(b) a stationary user who is looking around, e.g., while trying to orient themselves or looking for a landmark that was referred to in an instruction;
(c) different kinds of locomotion and transitions thereof, i.e., walking straight, walking around corners and going through doors; and
(d) the direction of doors, i.e., whether they need to be pushed or pulled.

The distinction between the first two activities is meant to bridge the gap between the physical world and the user’s current cognitive state. When integrated into an assistance system, it can give cues about how to proceed in helping users reach their destination. As an example, if a user is stationary and looking around for a long time during navigation, additional or different navigation instructions for the current step might be helpful. In combination with device placement information, assistance can be even more targeted by adapting the modality of new instructions, e.g., by providing audio instructions if the device is located in the pocket.

The remaining activities are more geared towards the idea of generating additional input for a PDR based indoor positioning system. When it is known that the user went around a corner or through a door, the positioning system can use this information to narrow down its hypotheses about the current location of the user. This, of course, requires a somewhat detailed model of the environment, especially for the use-case of the door direction detection. Here, we assume that some doors only open in one way, which allows the system to either confirm the user’s walking direction or limit the set of possible user locations. To integrate this information into a positioning system, particle filters [18] and HMMs [29] have proven useful in the past.

In contrast to existing approaches that rely on a single classifier to try to determine the user’s activity, we envision a system that consists of an ensemble of heterogeneous and hierarchical classifiers for specific use cases. As an example, a top-level classifier can detect whether the user is stationary or in motion, whereas a subsequent classifier differentiates between more precise sub-categories. Furthermore, if we can confidently make assumptions about the device placement, specialized classifiers can potentially improve activity detection accuracy in comparison to catch-all solutions that need to work for every placement.

The major advantage of this approach is the lower amount of classes that have to be distinguished at any point in time, making it easier to train classifiers. It also has the side-effect of being able to improve specific classifiers independently or even using different methods for the lower-level classification problems. One obvious drawback is, of course, the higher computational load, along with an increased memory demand, when multiple classifiers need to be run sequentially in order to make a decision. As we show below, on modern smartphones, this is not an issue in practice.

Our main contributions in this paper are as follows:

(1) We collect and describe a dataset containing fine-grained pedestrian navigation activities that exceed the generic classes found in other datasets.
(2) We design and evaluate an activity recognition approach that relies on hierarchical deep learning classifiers for these specific activities.
(3) We apply the approach to naturalistic log data from real users of a navigation app in order to gain insights into which device placements and activities occur during the course of a typical navigation session.

The remainder of the paper is structured as follows. In Section 2, we first describe the activity dataset specifically collected for this paper (Section 2.1) and then the proposed HAR methodology including the LSTM model (Section 2.2). We also briefly introduce two other datasets that serve as case studies (Section 2.3 and Section 2.4). Afterwards, the evaluation results are presented in Section 3.1. Finally, we apply the classifiers to experiment data (Section 3.2) and naturalistic usage data from our campus navigation app (Section 3.3) to check whether we can consistently find those patterns during actual usage. We discuss the findings in Section 4 and conclude the paper in Section 5.

## 2. Materials and Methods

In this section, we describe three datasets as well as the HAR methodology. The first dataset, which we call the Activity Logs Dataset, is used to train the classifier hierarchy, which is then applied to the others (Navigation Experiment Dataset and Naturalistic Dataset).

### 2.1. The Activity Logs Dataset

As established above, no existing HAR datasets fill our need with respect to smartphone-based pedestrian indoor navigation. Thus, for this study, three persons, each equipped with a Google Pixel phone, collected sensor data for various combinations of activities and device positions. The selection of sensors was as follows:

- The linear acceleration sensor is Android’s variant of the acceleration sensor that does not include the Earth’s gravity. It is useful for detecting motion along one (or more) of the device’s axes, be it periodic or sporadic.
- The gyroscope allows us to detect rotation and therefore—over a longer period of time—changes in device orientation. Typically, gyroscopes react much more quickly than magnetic field sensors but have the disadvantage of accumulating drift over time.
- The magnetic field sensor provides—in theory—the absolute orientation of the device with respect to the earth’s magnetic field. In practice, it is often too slow to react to sudden changes in orientation and can be disturbed by external influences, especially inside buildings.
- The proximity sensor was also active during data collection and is only used during data pre-processing but not for classification. It is located near the earpiece and detects if the front of the device is close to an object. We use it to estimate whether the device was held in the phone call position or put into the pocket.

As mentioned in the Introduction, we collected data for the following seven different activities, identified by uppercase labels used throughout the evaluation below:

- WALKING_STRAIGHT, LEFT_TURN and RIGHT_TURN cover three different kinds of regular locomotion on a level surface.
- STANDING_STILL and LOOKING_AROUND are two types of stationary behavior:
  STANDING_STILL simulates the case where a user is mostly not moving, e.g., when reading signage on a wall or instructions on their phone, whereas LOOKING_AROUND implies that the user is—while still mostly stationary—more actively looking at their surroundings, leading to increased upper body motion. The rationale behind distinguishing between these two activities is the pursuit of a more context-aware navigation assistance.
- THROUGH_DOOR_PUSHING and THROUGH_DOOR_PULLING as the final two activities are detected whenever a user encounters, opens and walks through a door that needs to be pushed or pulled, respectively. Detecting a door transition provides valuable information to the indoor positioning system and directly allows it to make fine-grained assumptions about the user’s position, decreasing its uncertainty. This is even more true if we can also correctly detect the orientation of the door, at least for cases where it only opens in one direction.

In line with much of the related work in the literature described above, we investigated four different device placements that cover most of the possibilities in a pedestrian navigation setting. Again, these are referred to by their respective labels below.

- Handheld, in front (IN_FRONT): This placement occurs essentially in every navigation session when the user actively interacts with the screen of the device. Additionally, this covers the periods where the user is holding the device in front of the body, following instructions and ready to interact at any moment.
- Handheld, swinging (IN_HAND_SWINGING): The second case is when the device is still held in hand, but not statically in front of the body, and rather swinging off the side in an outstretched arm. Here, the device movement is most decoupled from the user’s activity, imposing additional challenges for activity recognition.
- Handheld, phone call (IN_PHONE_CALL): This covers the case where the phone is held tightly and generally upright next to the user’s head, potentially picking up more of the upper body motion.
- Phone in pocket (IN_POCKET): This class of placements covers situations in which the phone is put away into a pocket, usually at waist-level. It has the advantage of being more tightly coupled to the user’s lower body movements but might increase the difficulty of distinguishing between some of the more granular activities.

**Figure 1:**
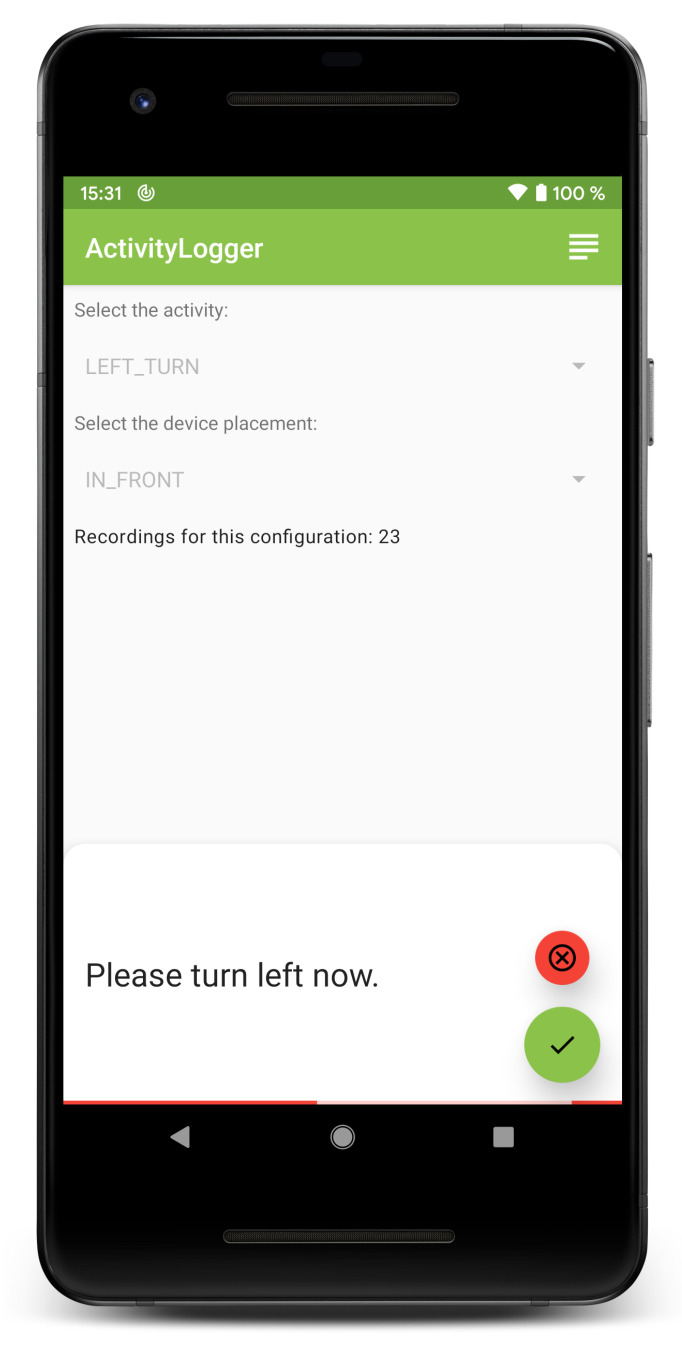
Screenshot of the data collection application while recording sensor data.

In total, this leads to 28 combinations of user activity and device placement. For data collection, a custom Android app was developed, allowing for easy selection of any of those combinations (see Figure 1). After selecting the activity and device placement and pressing the record button, the app displays the corresponding instruction and starts collecting sensor data. When the test person has finished performing the activity, they press the finish button in order to stop data collection. In case something goes wrong, the current data can be discarded by pressing the cancel button.

Data collection took place over the span of several days. The participants were instructed to record at least 20 trials for each combination of activity and device placement. Since the data are location-independent, there were no restrictions on where data collection should take place. To capture a realistic sample, there were also no detailed instructions on how exactly the device should be held during IN_PHONE_CALL or IN_HAND_SWINGING or in which pocket the device should be put.

Before working with the data, some pre-processing was necessary: Despite best efforts, some data were erroneous, e.g., impossibly short or long. The median duration for an individual logging session was 8.6 s, and half of all data fell into the range between 5.8 and 12.4 s. After visual inspection, a few more sessions were filtered out since they contained almost no acceleration even though their respective activity would require them to do so. Overall, 27 out of 1763 sessions were eliminated this way.

To create a clean dataset without artifacts stemming from handling the device during data collection, more pre-processing was done. For the device placements IN_PHONE_CALL and IN_POCKET, proximity sensor data were used to determine when the device was in its intended position, i.e., when the proximity was low—either due to the phone being in the pocket or near the person’s head. Afterwards, fixed amounts of 500 ms were cut off at the start and end of each session to account for the period where the device was put into position.

**Figure 2:**
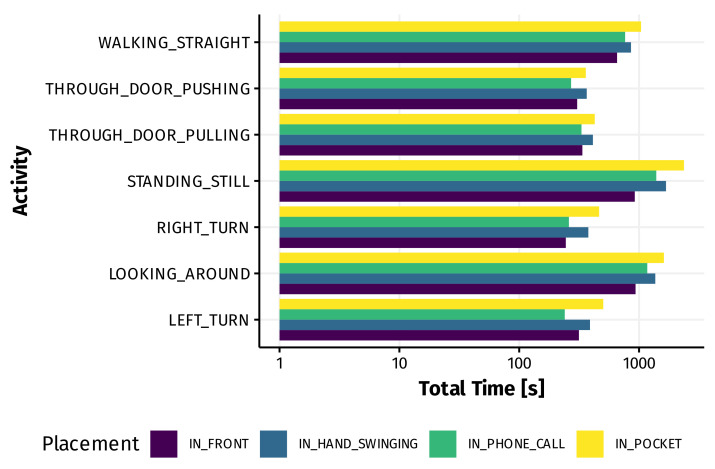
Amount of data for each combination of activity and device placement.

Gyroscope and linear acceleration data were sampled at 100 Hz, while the magnetic field sensor only updates at rate of 50 Hz. Therefore, its data were upsampled to match the other sensors. Finally, readings from the different sensors were aligned based on their timestamps, resulting in a nine-channel dataset (X, Y and Z for each of the three sensors used) consisting of more than 6 h of data. Figure 2 shows the amount of data for each of the 28 combinations after pre-processing. The dataset is available for other researchers; please refer to the Data Availability Statement below for more information.

### 2.2. Activity Recognition Methodology

**Figure 3:**
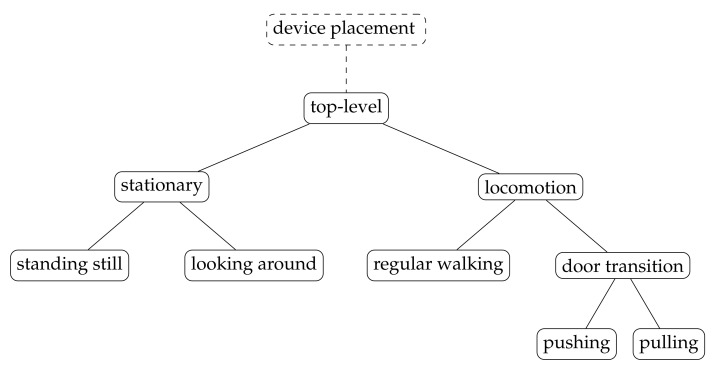
The hierarchical classification approach consisting of multiple binary classifiers arranged in a tree structure.

As described in the Introduction, we follow a hierarchical HAR approach to reduce the amount of classes each classifier has to be able to distinguish. The top-level classifier discriminates between stationary behavior (STANDING_STILL and LOOKING_AROUND) and locomotion (remaining activities). Subsequent classifiers determine the activity subtype, in the special case of door direction detection descending one additional level. All of these classifiers can either be general-purpose or conditioned to a particular device placement which has to be predicted first by an additional placement classifier (see Figure 3).

**Figure 4:**
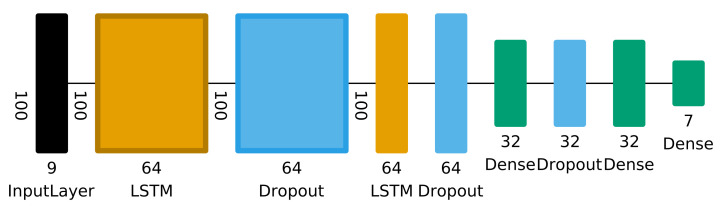
Schematic of the used neural network model. Hyperparameter optimization was used for the LSTM layer size (16/32/64) and Dropout (0.2–0.8). The size of the final dense layer varies depending on the number of classes needed for each classifier configuration.

For the individual classifiers, we rely on state-of-the-art deep learning methods. The classifier was built with the Keras and TensorFlow libraries and consists of two stacked LSTM layers that aim to extract temporal patterns in the data. Afterwards, fully connected layers process the output of the LSTM and lead to the final classification result (see Figure 4). This setup was chosen as as simplified version of the DeepConvLSTM method proposed in [10] since one of our design goals was to create a solution that is lightweight enough to be deployed on a mobile device and make predictions in real-time.

Hyperparameter optimization was performed for the Dropout rate and LSTM layer size, the latter affecting not only the system’s ability to accurately perform the classification task but also its performance. The search space for the optimal values consisted of the range 0.2–0.8 for the Dropout rate and the values 16, 32 and 64 for the LSTM layer size. For the optimization itself, the Python packages hyperopt and hyperas were used.

**Figure 5:**
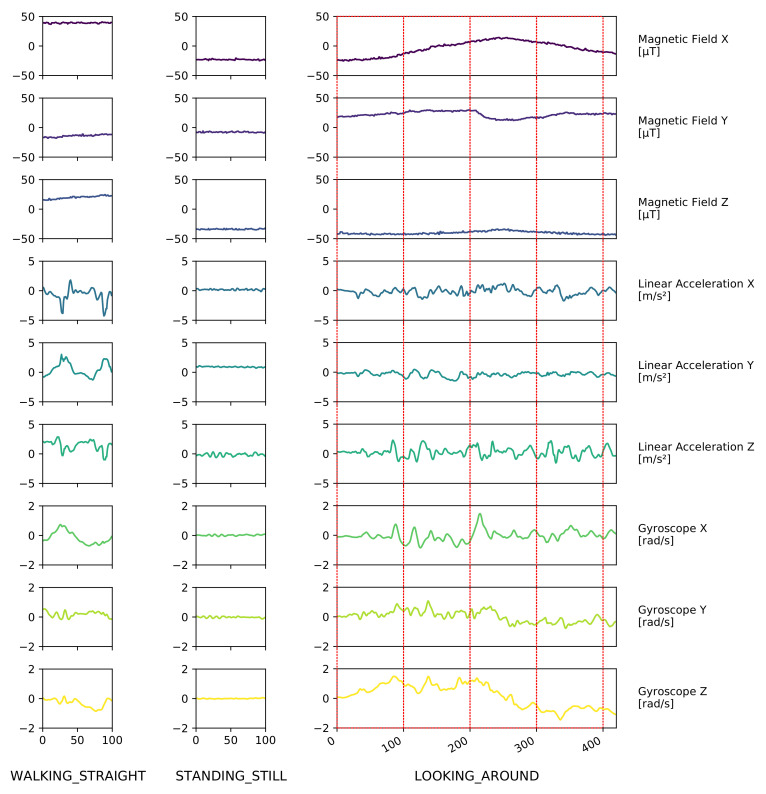
Examples of raw data for different activities collected in the Activity Logs Dataset. Red regions in the right-most graph illustrate how a stream of continuous data is split into 1-s windows consisting of 100 sensor readings each.

Since it is crucial in a real-time navigation setting to generate results in a timely manner, 1-s windows were used for classification. This corresponds to 100 sensor readings at a sampling rate of 100 Hz. Figure 5 shows these windows (red rectangles) in the context of their surrounding data as well as a few more examples for different activity classes, illustrating the range of patterns we encountered in the dataset.

### 2.3. The Navigation Experiment Dataset

After creating and evaluating the classifiers with the dedicated Activity Logs dataset, we also applied them to two other datasets collected under realistic navigation conditions. The first of them originates from a controlled indoor navigation experiment. Twelve test persons walked along three different routes across multiple floors of a university building (ranging between 250 and 300 m each). They were following the instructions of a navigation app and were accompanied by an experimenter who annotated the ground truth on specific locations. The test device—also a Google Pixel phone—recorded data from various sensors, including the ones described above which we need in order to perform activity recognition on this dataset.

In total, the experiment data span more than 2 h. With each individual run cut into segments that match the shape of the activity logs dataset, the resulting dataset consists of 7719 entries, each of those containing 100 sensor readings in 9 channels.

### 2.4. The Naturalistic Dataset

**Figure 6:**
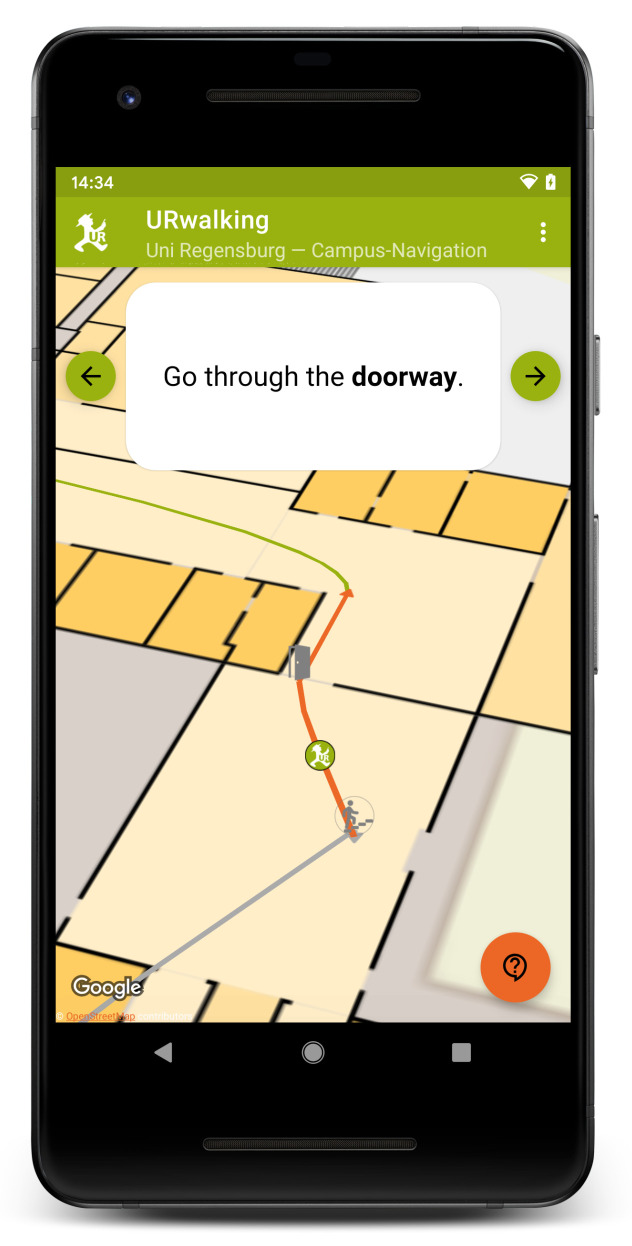
User interface of the app during navigation.

The final dataset used in this study consists of naturalistic log files collected from users of our navigation app for the Android platform. It was designed to provide map information and navigation assistance on our university campus and is available for free on the Google Play Store. In the app, users can request routes to arbitrary locations on the campus, which are divided into segments with corresponding text-based instructions and nearby landmarks. A work-in-progress indoor/outdoor positioning system tracks the user’s progress along the route. Figure 6 shows the app’s user interface during navigation.

When the app is first started, users are asked whether they’d like to anonymously share their usage data for research purposes. Provided that the user agrees, the supplied data include all the sensor data we need for this study, i.e., from accelerometer, gyroscope and magnetometer. Additionally, we receive information about the user’s location and route, their interactions with the app’s user interface and additional metadata.

Thus, far we have collected data from 1677 devices, totaling 13,541 sessions, over a span of more than two years. However, for this study, we are only interested in a subset of them that can be considered actual navigation sessions on the university campus. Previous studies have shown that the app is often used for looking up routes ahead of time and off campus, e.g., from their home or while riding the bus [30].

Therefore, the dataset is filtered heuristically to only include sessions originating on the campus, with the initial user position close to the start of the route. Additionally, very short (<1 min) and long (>30 min) sessions were excluded. The short sessions are in most cases an artefact of either user error (e.g., realizing the wrong destination was selected) or the app crashing unexpectedly while starting the navigation. Very long sessions can occur when the app is left running indefinitely without explicitly closing it or reaching the destination. The upper threshold was selected as it provides enough time to reach all locations on the university campus. Finally, to make sure an actual attempt was made by the users to reach their goal, we only include sessions where the built-in step detector registered at least 50 steps. After applying these restrictions, we receive a dataset of exactly 800 navigation sessions from 387 users.

## 3. Results

In this section, we first present the results of training hierarchical classifiers for the different activities described above. Afterwards, we show how the HAR approach can help in gaining insights into real-world datasets.

### 3.1. Classification Performance

For evaluation, the activity logs dataset was split into 80% training and 20% testing data. On the training set, stratified five-fold cross-validation was performed in order to avoid overfitting. Since the amount of data for the different activities was often imbalanced, custom class weights were set during training. Throughout the evaluation, DeepConvLSTM was used as a comparative benchmark representing a state-of-the-art HAR method.

#### 3.1.1. Device Placement

**Figure 7:**
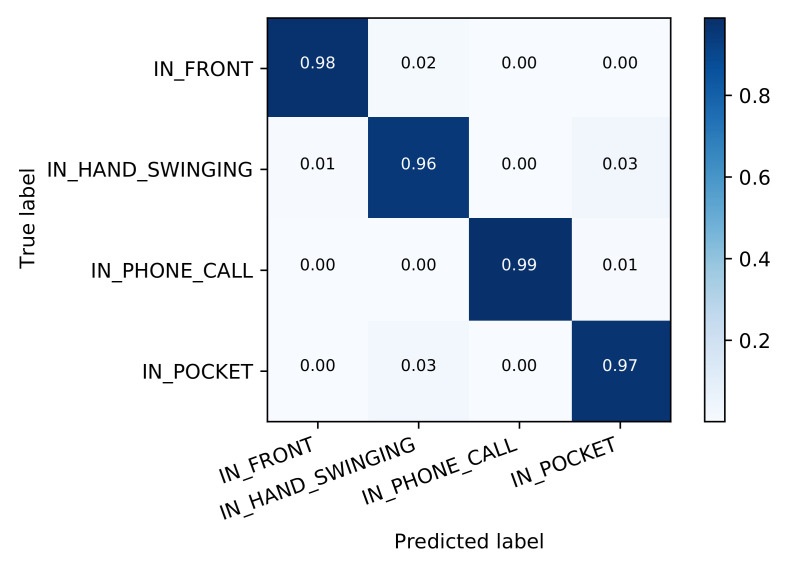
Classification accuracy for the device placement classifier.

Before looking at different types of activities, we used the methodology described above to train a classifier that determines device placement without knowledge about the current activity. This works very well, with an accuracy of 97.2%. In comparison, the DeepConvLSTM method reaches 98.0%. As can be seen in Figure 7, all classes are detected about equally well.

This result is somewhat expected since the four classes are quite distinct in respect to the device’s orientation and its degrees of freedom: While a device may mostly point downwards when sitting in a pocket and when swinging from the hand of the user, the latter case introduces much more acceleration in various directions. When held in front of the body, the device is about level or slightly tilted upwards, with some amount of freedom to move around, while, in a phone call situation, it is usually held upwards and more firmly against the head.

#### 3.1.2. A General Classifier for All Activities

To compare the hierarchical approach to a more traditional one, we first trained a single classifier for all seven activities. For this and the upcoming results, we initially report the results of a classifier that works without knowledge of the device placement. Afterwards, specialized individual classifiers are tested for which we assume to already know the device placement by running the corresponding classifier.

**Figure 8:**
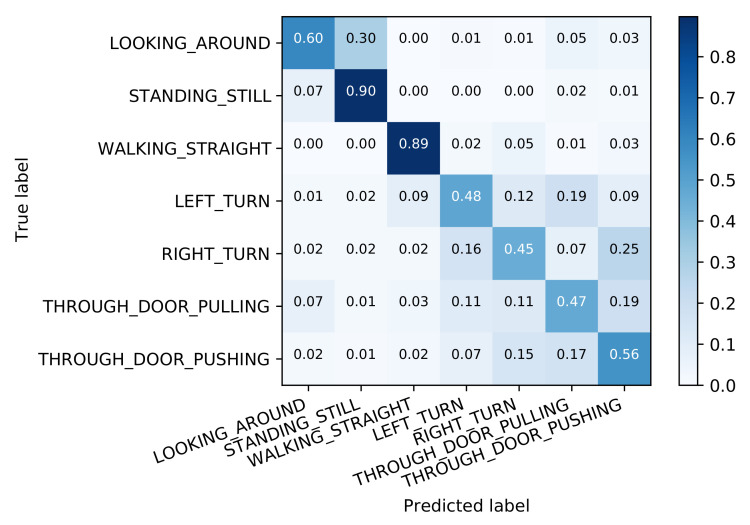
Classification accuracy for the all activities classifier.

The general classifier reaches an overall accuracy of 68.8%. While some classes such as STANDING_STILL and WALKING_STRAIGHT are detected quite reliably with an accuracy of about 90%, there is a lot of uncertainty with respect to turns or door transitions (see Figure 8).

These results are on par with those of DeepConvLSTM (68.6% accuracy), indicating that the comparatively low performance is not an issue with the trained model, but rather the challenging dataset.

Specialized versions for the different device placements do not improve the situation significantly: the best performance is achieved with the device held in front of the body (74.3% accuracy), while, during the other placements, we observe an accuracy between 68.4% and 69.7% (see Table 1, Column 1).

#### 3.1.3. Locomotion vs. Stationary Behavior

Since the general classifier does not perform well, we now turn our attention to hierarchical classifiers. First, we create and evaluate a top-level classifier that distinguishes between locomotion and stationary behavior.

The general classifier differentiates locomotion and stationary periods with an accuracy of 97.0% (DeepConvLSTM: 98.4%). With prior knowledge about the device placement, this value increases in some cases: with the device in front, we reach an accuracy of 98.7%, while the phone call configuration leads to a correct classification in 98.1% of cases. For the other two placements, no improvement could be measured with specialized classifiers compared to the general case (see Table 1, Column 2). Overall, these results are very good, allowing us to determine with a high likelihood whether the user is stationary or in motion.

#### 3.1.4. Types of Stationary Behavior

Once we know that the user is not moving, a secondary classifier can be used to differentiate between the two types of stationary behavior, i.e., STANDING_STILL and LOOKING_AROUND.

Overall, without knowing the device placement, we achieve an accuracy of 84.4% (DeepConvLSTM: 84.0%). Considering the fact that these two activities are quite similar, this is an encouraging result. Even more insight can be gained by analyzing the different device placements individually: if we know that the device is in front of the body, the activity can be correctly determined in 93.0% of all cases. This number drops to 84.9% and 78.7%, respectively, for the swinging hand and phone call cases. When the device is in a pocket, we achieve an accuracy of 88.8% (see Table 1, Column 3). These results show that the device is most likely to pick up the differing motion patterns in these two activities when it is held in front of the body.

#### 3.1.5. Locomotion vs. Door Transitions

Next, we try to build a binary classifier that can differentiate between regular locomotion and door transitions. For this end, we combine the activities THROUGH_DOOR_PUSHING and THROUGH_DOOR_PULLING into one class and WALKING_STRAIGHT, LEFT_TURN and RIGHT_ TURN into another. This poses the obvious challenge that locomotion is a part of any door transition, therefore we observe similar patterns in both classes.

Nevertheless, without knowing the device placement, we achieve an accuracy of 77.5% (DeepConvLSTM: 79.5%). Specialized classifiers for each of the device placements sometimes exceed this value, again with the IN_FRONT case performing best at 89.7% accuracy, followed by IN_HAND_SWINGING at 83.1%. IN_POCKET reaches 73.4%, while IN_PHONE_CALL trails behind at 70.3% (see Table 1, Column 4).

#### 3.1.6. Door Types

Finally, we try to build a classifier that is able to determine the direction of a door transition, i.e., distinguishing between the activities THROUGH_DOOR_PULLING and THROUGH_DOOR_ PUSHING.

Agnostic of the device placement, the classifier reaches an accuracy of 71.4% (DeepConvLSTM: 72.6%). Among the individual classifiers, IN_FRONT again performs well at 82.9%, only being surpassed by IN_PHONE_CALL at 86.5%. The other two placements reach 78.9% (IN_HAND_SWINGING) and 62.6% (IN_POCKET) (see Table 1, Column 5).

#### 3.1.7. Performance on Actual Devices

To judge whether the trained models can be deployed in real-world applications, their performance was tested on an actual Android device. For both our approach and the DeepConvLSTM benchmark, a worst-case scenario was selected where the hyperparameter optimization chose the largest LSTM size.

First, the models were converted to the TensorFlow Lite (TFLite) format, which is specially optimized for mobile and low-power devices. A Google Pixel phone running Android 10 served as the test device. Originally released in 2016, it can nowadays be considered a good representative of a mid-range Android device. The evaluation was performed with help by the TFLite Model Benchmark Tool which allows to repeatedly run inference on a supplied TFLite model with random inputs. One hundred runs were performed for each model, using 4 CPU threads.

**Figure 9:**
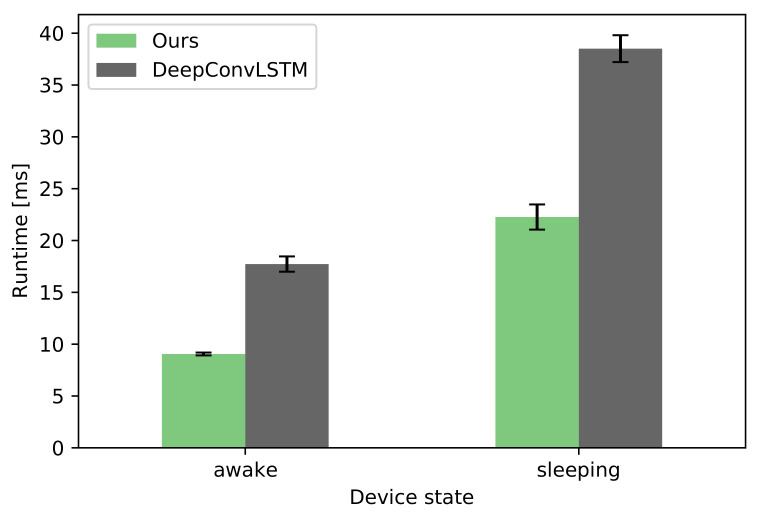
Comparison of runtimes of a single inference on a Google Pixel phone.

When the device is awake (screen turned on), running inference a single time on average takes 9.05 ms for our model, whereas the more complex DeepConvLSTM model takes 17.7 ms to compute. With the screen turned off and the device in a low-power mode, the times roughly double to 22.3 and 38.5 ms, respectively (see Figure 9). The results show that both models are very much applicable in pedestrian navigation situations where near real-time classification is needed once per second—even when the device is resting in the pocket and sleeping. The temporal constraints also allow for running multiple of these classifiers in sequence, as required by the envisioned hierarchical model described above.

### 3.2. Applying Classifiers to the Navigation Experiment Dataset

After successful training and evaluation, we can now demonstrate a proof-of-concept on how the ensemble can be applied to an actual navigation dataset. While during the experiment no detailed ground truth was collected with respect to user activity at specific points in time, we can check whether the classifiers work correctly by looking at the class distribution.

During the experiment, test persons needed to interact with the navigation app in regular intervals and therefore kept the device in their hand, mostly in front of their body. The results of the device placement classifier support this: Of the 7719 data points, 6408 or about 83% are classified as IN_FRONT, another 345 (4.5%) as IN_HAND_SWINGING. However, 960 or 12.4% are misclassified as IN_POCKET, whereas the remaining six cases are erroneously detected as IN_PHONE_CALL.

**Figure 10:**
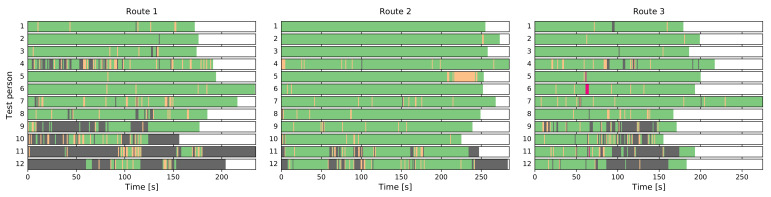
Results of the device placement classifier for the three test routes and 12 participants each (green, IN_FRONT; yellow, IN_HAND_SWINGING; red, IN_PHONE_CALL; grey, IN_POCKET).

Figure 10 shows the results for each of the three test routes and 12 participants in more detail, with color-coded device placements along the course of each test run. Overall, IN_FRONT (green) is predominant, but especially in Routes 1 and 3 misclassifications (grey periods) occur for some test persons.

In this particular case, the DeepConvLSTM solution performs better and—presumably not least due to its larger size—is more able to generalize to this new dataset, mistaking only 182 of the 7719 samples as IN_POCKET.

Turning our attention to the top-level activity classifier, it detects locomotion in 5665 cases (73.4%) while the remainder is considered stationary. This meets our expectations since the test persons were mostly following the navigation instructions, but had to sometimes stop in order to re-orient themselves. Additionally, the dataset includes the moments at the beginning of the experiment runs where data collection was already running but the test persons had yet to move.

**Figure 11:**
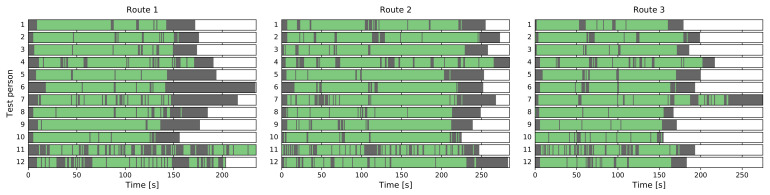
Results of the locomotion/stationary classifier for the three test routes and 12 participants each. (green, locomotion; grey, stationary).

Detailed results for the locomotion/stationary classifier for the whole dataset can be seen in Figure 11. We consistently see the aforementioned stationary periods at the beginning and end, as well as shorter pauses scattered throughout the runs. In many cases, they coincide temporally across multiple users (see, e.g., Route 3), corresponding to the same location on the test route.

After identifying the periods where the user was likely stationary, the subsequent lower-level classifier can be applied. The 2054 stationary cases are not quite evenly split into 40.3% (LOOKING_AROUND) and 59.7% (STANDING_STILL). In this circumstance, we would expect such an outcome since the test persons had to combine information on their phone screen with an oftentimes unfamiliar environment.

### 3.3. Finding Behavioral Patterns in the Naturalistic Dataset

After having verified their utility in an experimental dataset, we now apply the best-performing classifiers to the naturalistic navigation log dataset from 387 users introduced in Section 2.4. This allows gaining novel insights into behavioral patterns during pedestrian navigation. Since these data were not collected in a supervised environment and therefore no ground truth about the user’s position or activity exists, the analysis cannot focus on individual navigation sessions, but rather paint a general picture about user behavior and changes thereof over time. In particular, we investigate

(a) how device placement changes over time in a typical navigation session;
(b) patterns in the distribution of stationary and locomotion periods; and
(c) the correlation of device placement and activity.

#### 3.3.1. Changes in Device Placement over Time

Based on earlier studies, we know that users tend to carry their device in their hands for the majority of time when navigating [30]. By applying the device placement classifier to naturalistic navigation data, we can further investigate how exactly this changes over time during a typical navigation session. As detailed above, for all upcoming analyses we use the filtered dataset, containing only sessions during which we can confidently assume an attempt at navigating towards the destination was made.

After merging the sensor data of each session and splitting it into 1-s chunks, we run inference in order to get a vector of classification results. Since the length of the navigation sessions varies, each one is divided into 100 evenly-sized parts for which the prevalent device placement is determined. This discretization allows us to compare the changes over time for differently-sized sessions.

**Figure 12:**
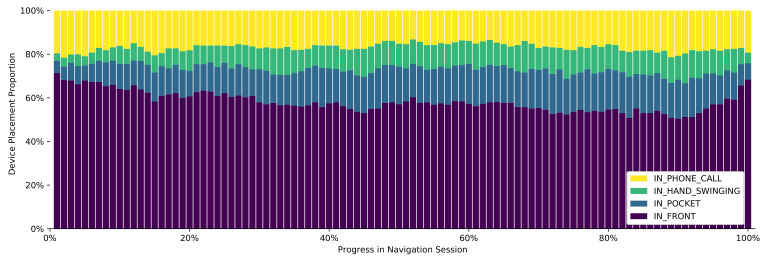
Distribution of device placement over the time of a navigation session.

The results of this procedure are displayed in Figure 12. As expected, IN_FRONT is the most prevalent placement overall. Over time, it decreases from an initial value of 70.5% down to a minimum of 51.9%, while IN_POCKET increases from 5.8% to a maximum of 20.9%. Near the end of a navigation session, the share of IN_FRONT sharply increases again to roughly the initial value.

#### 3.3.2. Stationary and Locomotion Periods

This next analysis focuses on the time spent in motion vs. being stationary (and its subcategories). After preparing the dataset in the same fashion as above, two classifiers are applied in sequence: First, the top-level classifier distinguishes between locomotion (55.9% of time) and stationary (44.1%) behavior. The latter is subsequently classified again, resulting in 63.7% LOOKING_AROUND and 36.3% STANDING_STILL.

**Figure 13:**
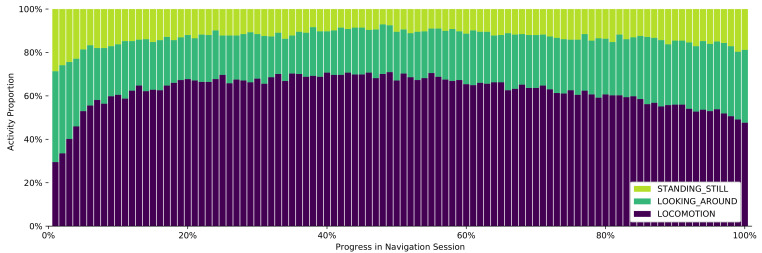
Locomotion and stationary behavior over the time of a navigation session.

After discretizing the result, we see that these numbers are not constant but change greatly over time (Figure 13). Initially, the stationary modes are outnumbering locomotion by far, indicating that users first have to understand the initial instructions or orient themselves on the map. Soon, however, locomotion takes over as the most prevalent activity, staying above 60% for most of the time. The remaining percentage is shared by the two stationary modes, with LOOKING_AROUND always being more common than STANDING_STILL. Near the end of the session, locomotion decreases and the two top-level behaviors are almost equally often observed again.

#### 3.3.3. Correlation between Device Placement and Activity

Finally, we investigate to what extent device placement and activity are correlated with each other. Prior knowledge about this allows a system to make certain assumptions e.g., when deciding which assistance method to employ in guiding a user.

The relation between device placement and activity is, in fact, significant, as confirmed by a chi-square test, $\chi^{2}$(6, N = 169,040) = 10,309.559, *p* < 0.001.

**Figure 14:**
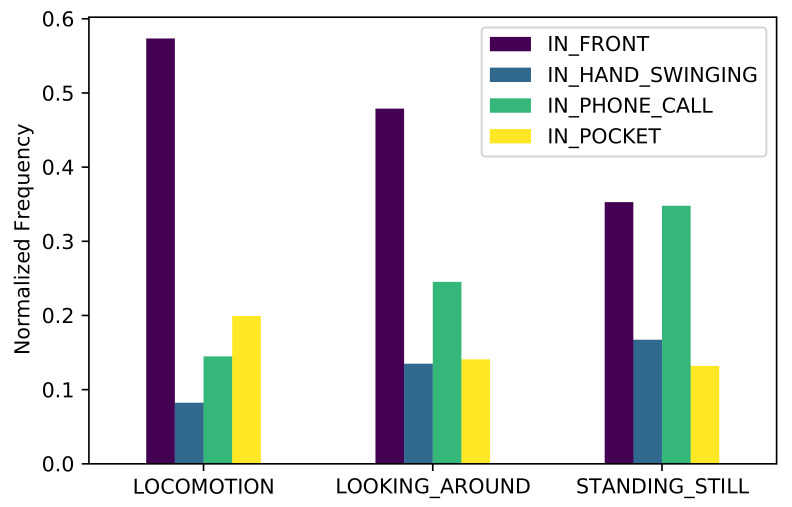
Normalized frequency of each device placement during the main activities.

Figure 14 shows the normalized occurrence rates of the different device placements for each activity. As we already know, IN_FRONT is the most common placement overall. The second-most common device placement during locomotion is IN_POCKET, while during stationary periods it is less prevalent. This can be explained by the users stopping and pulling their device out of the pocket when they do not know how to proceed towards their destination.

## 4. Discussion

The results in Section 3.1 show that it is very reliably possible to distinguish between the four device placements in our training dataset. This provides important information for targeted assistance and allows the system to adapt to different situations. When applied to the experiment dataset, the classifier erroneously detected the IN_POCKET case too frequently for some users. A possible explanation for this is a different (or lack of) calibration of the device sensors. The experiment runs in question (7–12) were performed right after each other. Another option is that, since our four classes simply do not cover all the possible device placements, this inevitably leads to misclassification at some point.

Compared to the experiment dataset, the share of locomotion is much lower in the naturalistic dataset. This is not unexpected, since during the experiment the participants had a concrete task of walking along a predefined test route, with an experimenter nearby to make sure they fulfill their task. In a real-world scenario, one can imagine many reasons a user would stop following the route for a while or cancel the navigation session altogether.

Determining the user activity when no movement is detected works quite well, especially when the device is held in front of the body, which—as we demonstrated—is the most common mode. This information can inform the decision of the navigation system whether or not to provide further navigation assistance, i.e., by referring to more or different landmarks for orientation. During locomotion, the detection of door transitions vs. regular walking motion is also quite reliable, with results that are similar to our previous ones based on a different dataset and methodology [31]. The distinction between different kinds of doors, i.e., between pushing or pulling, however, is not as easy to perform. Presumably, the motion patterns are simply too similar for most device positions. The worst performance in this context is observed when the device is located in the pocket and therefore most tightly coupled to the body of the user.

In the literature, sophisticated HAR methods achieve up to 95% accuracy on some datasets [9]. Except for the top-level classifier, our results range substantially lower than that figure. However, the comparison with DeepConvLSTM shows that the reason for this is not our model performing badly (in fact, we outperform it sometimes), but rather the more challenging dataset containing classes that cannot be distinguished clearly at all times due to overlapping motion patterns.

The hierarchical approach proved advantageous especially in the cases where one of lower-level classifiers for a specific device placement greatly outperforms its generic counterpart, e.g., the dedicated IN_FRONT classifier for the stationary types or the locomotion/door distinction or the door direction classifier specialized for the IN_PHONE_CALL placement.

## 5. Conclusions

In this paper, we present an approach for HAR centered around fine-grained activities that occur during smartphone-based pedestrian navigation. We considered multiple device placements and collected an annotated dataset for many activity/placement combinations. We used this dataset to train a hierarchy of ML classifiers and evaluated it against the state of the art. Afterwards, we applied the method to real-world navigation log data in a case study and demonstrated how it can help in gaining insights into navigation behavior. While the results lead to believe that the trained models can generalize to unknown data quite reliably, the classification can be even more robust with additional labeled data.

One question every HAR system has to consider is how to handle motion data that do not fall neatly into one of the predefined categories. The proposed hierarchical approach has the advantage of being able to extend or re-train parts of the system in order to adapt to different classification needs. While we did not cover any means of level transitions such as elevators, staircases or escalators in this paper, this can easily be included if required by the environment the system is deployed in.

How to integrate these results into a pedestrian navigation system in order to provide better assistance in the form of instructions is another question to be addressed in future work. In particular, this entails the problem of how a positioning system could use this information in order to improve its accuracy. For now, we ensured that the proposed system can be deployed on actual devices and perform classification in near real-time, thereby enabling navigation assistance for pedestrians to adapt to user behavior.

## Figures and Tables

**Table 1:** Classification accuracy for different configurations and scenarios.

Device Placement	1 General	2 Loco. vs. Stat.	3 Stat. Types	4 Loco. vs. Door	5 Door Types
unknown	68.8	97.0	84.4	77.5	71.4
IN_FRONT	74.3	98.7	93.0	89.7	82.9
IN_HAND_SWINGING	68.8	97.0	84.9	83.1	78.9
IN_PHONE_CALL	69.7	98.1	78.7	70.3	86.5
IN_POCKET	68.4	97.0	88.8	73.4	62.6

## Data Availability

The HAR data set presented in this study is available at https://ai.ur.de:81/indoor-positioning/pedestrian-navigation-har-dataset (accessed on 6 May 2021).
